# SAFEvR MentalVeRse.app: Development of a Free Immersive Virtual Reality Exposure Therapy for Acrophobia and Claustrophobia

**DOI:** 10.3390/brainsci14070651

**Published:** 2024-06-27

**Authors:** Marcel-Alexandru Gaina, Stefan-Vladimir Sbarcea, Bianca-Stefana Popa, Bogdan-Victor Stefanescu, Alexandra-Maria Gaina, Andreea-Silvana Szalontay, Alexandra Bolos, Cristinel Stefanescu

**Affiliations:** 1 Psychiatry, Department of Medicine III, Faculty of Medicine, Grigore T. Popa University of Medicine and Pharmacy of Iasi, 16 Universitatii Street, 700115 Iasi, Romania; andreea.szalontay@umfiasi.ro (A.-S.S.); alexandra.bolos@umfiasi.ro (A.B.); cri.stefanescu@umfiasi.ro (C.S.); 2 Institute of Psychiatry “Socola”, 36 Bucium Street, 700282 Iasi, Romania; 3 The Association of Integrative Psychotherapy and Clinical Psychology, 700469 Iasi, Romania; 4 Faculty of Computer Science, “Alexandru Ioan Cuza” University, 700483 Iasi, Romania; stefansbarcea@yahoo.com (S.-V.S.); popabianca945@yahoo.com (B.-S.P.); 5 Faculty of Medicine, University of Medicine and Pharmacy “Grigore T. Popa” Iași, 700115 Iasi, Romania; 6 PhD Department, University of Medicine and Pharmacy “Grigore T. Popa” Iași, 700115 Iasi, Romania; alexandra-maria.popescu@d.umfiasi.ro

**Keywords:** virtual reality exposure therapy, self-exposure, gamification, acrophobia, claustrophobia, transdisciplinary research and development, free VRET, Metaverse

## Abstract

**Background:** Specific phobias impact over 400 million people worldwide. Digitalizing mental health could alleviate the burden. Still, although the corporate-driven Metaverse is expanding rapidly, there needs to be more momentum in harnessing virtual reality exposure therapy uptake. **Objective:** This study aims to conceptualize, develop, and deploy a free Virtual Reality Exposure Therapy (VRET) application specifically designed for treating acrophobia and claustrophobia. This pilot study, which holds the promise of a future where mental health is more accessible and effective, explores the feasibility of leveraging transdisciplinary collaboration among specialists to create a safe, accessible, and effective VRET solution. **Methods:** We conducted a Delphi heuristic approach involving bioethicists, neuroscientists, and tech developers. Second, we reviewed the existing psychological theories and therapeutic strategies for addressing phobias in VR. Third, we conceptualized a thematic analysis-derived framework for a safe, adaptive-gamified free exposure to virtual reality acrophobia and claustrophobia (SAFEvR ACT). Finally, we provide an overview of the iterative improvements made during 12 workshops and 76 weekly briefings on developmental implementations. **Results:** We developed the SAFEvR ACT into a proof-of-concept application freely deployed on the MentalVerse app platform. Our safety-focused approach can benefit from prevalidation perspectives within future randomized control trials. **Conclusions:** The resulting application derived from the SAFEvR ACT framework represents a blueprint to counter the current lack of iVR mental health uptake by offering a free VRET alternative. Future research should aim towards developing similar free platforms to lessen mental health burdens and gather quantitative data. We conclude with a call to action to researchers to fine-tune our current approach and take a stand for free digital mental health within MentalVeRse.app.

## 1. Introduction

### 1.1. Background

Specific phobias affect over 400 million individuals worldwide [1]. Acrophobia and claustrophobia are two common distressing anxiety disorders that can cause suffering and functional impairment, compromising a person’s quality of life [2]. Acrophobia, commonly regarded as fear of heights, incapacitates up to five percent of individuals, always requiring psychotherapeutic intervention, as correlated apprehension overshadows patients’ quality of life beyond specific exposures [3,4,5]. Visual height exposure intolerance burdens as much as one-third of the general population with anxiety and distress, half of which require psychotherapeutic interventions [6].

Through its universal prevalence, visual height-related postural imbalance represents one of the few mental health burdens to have been widely destigmatized. This aspect derives from its commonly relatable character, regarded empathically by the general population. These are viable arguments for targeting this specific nosology when conceptualizing intervention models using immersive virtual reality (iVR) exposure [7]. 

Although widely prevalent, the interwoven autonomic archaic response triggers height exposure and symptomatic heterogeneity [8], leading to the contraction of anti-gravitational muscles [9], result in postural control system and oculomotor visual function impairments [10]. However, the aforementioned autonomic response is not directly proportional to the sensitive intensity of the phobic trigger but is primarily modulated by an irrationally perceived threat [11]. 

Claustrophobia, or the fear of confined spaces, affects approximately seven percent of the population [12]. Phobias often associate with significant avoidance behaviors, such as refusal due to impairment in undertaking everyday activities, and therefore, have essential social, occupational, and even health consequences concerning a lack of adherence towards screening painful medical procedures [13]. Acrophobia and claustrophobia can cause extreme avoidance of places or situations associated with a fear of dying, losing control, or being trapped [14].

### 1.2. State-of-the-Art Virtual Reality Exposure Therapy (VRET)

High-end iVR delivered through a dedicated stereoscopic head-mounted display (HMD), low-end immersive iVR content delivered through a smartphone-powered iVR cardboard headset, and computer-generated bi-dimensional display content are all considered VR. There is an urge for a specific MeSH (Medical Subject Heading) to clarify the differentiation of exposure types within the currently published literature [15].

Another manner of indexing iVR technology is according to the Extended Reality (XR) criteria, which includes iVR and Augmented Reality (AR)—a form of immersion that overlays elements on the user-perceived real-world environment [16,17], while Mixed Reality (MR) combines iVR and AR capabilities to facilitate seamless interactions [18].

The Metaverse represents the current flagship of immersive technologies, including XR, gaming, and social media, therefore representing a double-edged tool in mental health through its potential. Metaverse development should be shaped towards mental health advocacy [19]. 

iVR encompasses HMDs and motion-tracking systems and generates three-dimensional iVR environments that can be dynamically manipulated in real time by surrounding the user’s senses. iVR is promising for acrophobia and claustrophobia exposure therapy, which have a high reliance on the concept of presence [20,21]. iVR technology allows a novel controlled exposure that provides the possibility of confronting the phobia in a safe setting and achieving clinically relevant results [22], with evidence showing substantial improvements in therapeutic outcomes [23]. iVR has been extensively researched beyond specific phobias, and reports have validated a panoramic impact on both stress-related and chronic anxiety disorders [24,25]. Its effectiveness was also evaluated in treating anxiety related to minimally invasive medical procedures such as dentophobia [26] and trypanophobia and endoscopic medical procedures such as colonoscopy [15,27], hysteroscopy [28], and arthroscopy [29]. 

VRET undoubtedly delivers an advantage to practitioners compared to traditional exposure, as it is sometimes almost impossible to fine-tune exposure levels in real-world scenarios [30]. VRET’s potential resides in its ability to create personalized exposure sanctuaries for PTSD [31,32], or training grounds for fundamental research models [33,34,35]. The potential of virtual reality is proportional to an individual’s immersion level within the virtual environment, as presence is indispensable for VRET efficiency [36,37]. Presence inception is represented by the user’s perception of acceptance of the virtual environment, whereas the level of immersion is positively correlated with the cumulative effect resulting from the quality of sensorial information delivered through an HMD [15].

The use of consumer VRET to treat these phobias has been shown to be effective for acrophobia and claustrophobia, as well as demonstrating efficacy with the general treatment of specific and complex phobias [38]. A well-established literature body exists on exposure therapy that demonstrates its effectiveness in treating acrophobia and claustrophobia, showing that VR-based exposure therapy can also reduce these individuals’ anxiety. iVR adds extra levels of immersion to the treatment and can augment it with videogame-like elements that help keep the patient engaged and adhere to the therapeutic program by adding motivational factors to the exposure procedures. CBT remains the primary treatment for anxiety disorders, including those of the specific phobia subtype, given its robust evidence base [39]. Nevertheless, iVR technologies can be beneficial adjuncts, especially when traditional therapies are challenging to access [40]. Overall, using iVR to treat acrophobia and claustrophobia demonstrates the need to step forward and utilize this therapy as a means to offer distributive justice-derived treatments [41].

### 1.3. Current Limitations of VRET Mental Health Uptake State

The COVID-19 pandemic acted as a catalyst that shifted perspectives of digital therapeutics beyond telemedicine [42,43,44]. Currently, freely available dedicated VRET applications for acrophobia or claustrophobia treatment are either not scientifically validated or developed for clinical use [37,44], or share a technological bias of being delivered through a smartphone-powered interface, such as the *Easy Heights* application [45]. 

However, commercial interests primarily drive current VRET applications, and most of them need more rigorous scientific development and evaluation, affecting credibility [46]. To address these challenges, the design of future iVR applications should prioritize safety and comfort, address individual therapeutic goals, and be fully engaging to promote self-exposure [21] and even serve as a platform for human-delivered psychotherapy [47]. In addition, future research should focus on increasing the usability of iVR to integrate it efficiently within the workflow of clinicians [42], and to provide experience of VRET’s effectiveness to familiarize and adopt the intervention [48].

However, a significant concern that lacks adequate therapeutic counterbalancing is the impact of current technological disruptions on mental health. In particular, widespread access to technology has not been accompanied by sufficient mental health advocacy, leaving the detrimental effects of technological exposure unaddressed [49]. 

Although mobile phones, smartphones, and TV have advanced rapidly, a handful of relevant therapeutic platforms still focus on mental advocacy and access to these technological devices’ widespread availability within the general population. The *REThinkEMOTIONS* Project offers a new perspective regarding the autonomous delivery of psychotherapy in online environments. Bridging CBT interfaces involves developing and validating new psychometric dimensions [50] to facilitate applications for managing distress [51] or to address vulnerable pediatric populations through flexible interventions [52,53]. 

Although consumer iVR technology is widely available and affordable, the pressing absence of freely available VRET platforms persists [54,55,56,57,58,59,60,61,62,63,64]. On the other hand, the technology focuses on direct user benefits such as entertainment, which is likely because the current VRET technology development is profit-based, resulting in unethical practices such as preferential algorithms similar to those utilized on social platforms. Given recent events and increased public awareness, the US Congress has started pressuring such companies. However, coercive measures to curb this problem have yet to be introduced. For the most part, the development of iVR technology has been neglected from a mental health advocacy perspective. Hence, while research concerning technology is actively being conducted in various ways, from public polls to government-sponsored studies, there have been no efforts from mental health advocacy communities to combat the evident problems with technology’s negative impact on mental well-being [65]. The small progress in mental health advocacy was represented by offering an interface for those with psychological vulnerabilities that impairs social exposure [66] and the promise of improving health literacy and lowering destigmatisation [67]; the negative impact on the developmentally vulnerable adolescent population amplifies social interaction deficits while lowering self-esteem [68,69]. At the same time, the highly immersive platform turns to increased gaming experiences at the cost of reducing the mental health quality of adolescents by more than a third [70]. No free mental health clinics exist within a corporate profit-driven Metaverse [71]. 

Therefore, there is a need to engage iVR developers, researchers, and healthcare professionals to collaborate on developing inexpensive, evidence-based iVR therapies to turn iVR into a mainstream therapeutic approach. Still, the limited VRET uptake might be both related to and impaired by the quantitative-driven current state-of-the-art literature that favors more substantial evidence bases of current intervention through replication while giving little chance for innovative research endeavors to penetrate the scientometric flow [72]. 

### 1.4. Objectives

The present study intends to address these deficiencies by designing an innovative VR-based systemic therapeutic protocol tailored to overcoming acrophobia and claustrophobia: the Safe Adaptive-Gamified Free Exposure (SAFEvR) protocol. 

The main question addressed by this research is:

*What is the shortest path towards building a safe, adaptive-gamified, freely accessible virtual reality exposure therapy application for acrophobia and claustrophobia*?

To answer this question, the specific objectives of this research are as follows:

- First objective: Heuristically design a conceptual framework and translate it to a safe self-exposure VRET application product for treating acrophobia and claustrophobia;
- Second objective: Further incorporate the principles of adaptive gamification and personalized psychotherapeutic prompts to enhance iVR application user engagement;
- Third objective: Assess SAFEvR’s efficacy in reducing acrophobia and claustrophobia symptoms through Delphi transdisciplinary heuristic workshops, gathering expert feedback while emphasizing user safety;
- Fourth objective: Deploy the SAFEvR ACT MentalVerse.app on the MentalVerse.app platform to make it accessible and scalable.

## 2. Materials and Methods

Using a Delphi research and development approach, we conducted 12 workshops that involved all ten specialists mentioned above between October 2022 and March 2024 in testing, answering feedback questionnaires, and discussing iterative improvements. The resulting thematic analysis was discussed within 76 weekly in-person meetings or video briefing conferences involving ThinkThank members; these members were responsible for implementing the aspects discussed during workshops within the application. The ThinkThank members were the two software developers and principal investigators. A fourth member, B.V.S., ensured the up-to-date state of the WhatsApp (WhatsApp LLC, Menlo Park Menlo Park, United States of Amreica, Version 2.24.9.78) group, the GitHub (Microsoft Corporation, Redmond, United States of America, Redmond, United States of America, GitHub Enterprise Server Version 3.5.3 for MentalVerse.app and SAFEvR enterprise accounts), and Trello (Atlassian, Sydney, Australia, Version 2024.5.0) improvement checklists. The thematic analysis aimed to evaluate improvements towards the consumer-level risk of a VRET acrophobia and claustrophobia application, balancing psychotherapy, safety, and accessibility with technological interoperability, modularity, and user-centered design through an adapted therapeutic–digital interface. It aimed to gather and implement feedback from all stakeholders on the application’s design, functionality, and therapeutic content and postpone workshops until iterative commonly agreed feedback was implemented within the refined app version.

### 2.1. Transdisciplinary Bioethicists–Mental Health–Tech Development Approach

Harness’s transdisciplinary expertise combines insights from neuroscientists, iVR developers, user interface designers, and ethical advisors to develop an engaging and therapeutically effective VRET application (Table 1). 

### 2.2. Conceptualization of the SAFEvR ACT Framework

The SAFEvR protocol should facilitate patient adherence by balancing the exposure within a virtual sanctuary that aims to be vivid enough to immerse the patient and facilitate phobic desensitization progress but secure enough to ensure compliance and progress while following the extended framework described in Table 2.

### 2.3. State-of-the-Art Perspective Regarding Existing VRET Limitations

The current guidelines for incorporating a transparency framework are crucial for promoting user trust and experience. They focused on data sovereignty and usability improvements to optimize the VRET app design [73,74], as shown in Table 3.

Developing a trustworthy user interface design for a VRET app is vital for user experience and application efficacy. Critical elements for reliable design include interface design, feedback provision, and usability enhancements. These elements were extracted from the latest ISO Ergonomics of Human–System Interaction, Part 820: Ergonomic guidance on immersive interactions, including augmented and iVR [89] as listed in Table 4.

## 3. Results

SAFEvR ACT: a blueprint for a Virtual Reality SAFEvR (Safe Adaptative-gamified Free Exposure) ACT (Acrophobia and Claustrophobia Therapy) application walkthrough is available within Supplementary Video S1. and also at https://youtu.be/Z036SxBFq1E (accessed on 24 June 2024).

This section presents the main findings of the heuristic development workshops. At the same time, since the framework’s inception, the algorithm always focused on safety prioritization, from fine-tuning the initial application model to deploying the current version on the https://MentalVerse.app platform (accessed on 24 June 2024). The current version is compatible with both Windows and Android platforms, requiring a dedicated HMD; we recommend Oculus Quest 2 (Reality Labs, Meta Platforms) as a minimum requirement. The heuristic-driven implementations follow the SAFEvR concept, starting with the safety and ethical aspects (Table 5), followed by the adaptive gamification aspects (Table 6). 

The Supplementary Materials reveal the implementation aspects of the freely accessible user interface (Table S1); furthermore, a systematized table synthesizes the embedded psychotherapist voice guidance prompts that are triggered by the user’s contextual behavior during exposure (Table S2).

## 4. Discussions

Our transdisciplinary Delphi development model combines gamified adaptive strategies with exposure therapy experiences for both acrophobia and claustrophobia. A customizable and flexible IVR system will address users’ preferences and needs while developing a feasible guide for future VRET applications.

The SAFEvR ACT will remain a free-to-use application, prioritizing safety, engagement, and therapeutic efficacy. The purpose of this system is to decrease the mental health burden by building the confidence of people living with a phobia and to alleviate their suffering.

### 4.1. Neurologist VRISE Mitigation Heuristics

VR exposure may cause sensory overload [106,107], especially in susceptible individuals such as critically ill patients, where it might exacerbate their already enhanced sensory processing sensitivity [108]. For example, the experience might induce visually induced motion sickness (VIMS) characterized by nausea, dizziness, and disorientation, mainly containing sensory conflicts between the visual and vestibular systems [94,106]. In addition, lag or delay between virtual and real movements might decrease the sense of presence, impacting the user’s immersion and reducing the therapeutic benefit of iVR [109]. Colocation issues, such as when the user’s limbs are not aligned with the virtual ones depicted by controllers, can reduce the immersive benefit of VR, which is especially concerning for clinical pain management [110]. VR, where a person can feel embodied as an avatar, might not work for people with high proprioceptive acuity or aged individuals with more difficulties experiencing virtual embodiment in the first place [109]. Noise, light sources, or objects from the real world may also disrupt the sense of presence, thus decreasing the immersive quality [109]. Simulator sickness, a common adverse effect of prolonged iVR exposure that is similar to VIMS, is clinically expressed as a syndrome encompassing nausea, dizziness, headache, fatigue, and other symptoms, which might be more severe in older people and the Parkinson’s disease population [111,112]. Future research aims to investigate the role of iVR in assessing the risk of falling in Parkinson’s disease patients [113] and balance in the general population [114]. These symptoms have been thought to be caused by sensory conflicts and the reweighting of sensory signals, impacting perceptual estimates and potentially amplifying the severity of immersive cybersickness, especially in vulnerable patient situations, such as after undergoing surgical interventions [115]. Thus, despite these enormous therapeutic promises, considering these factors is imperative to mitigate adverse scenarios. A high correlation between the level of immersion and an increase in anxiety signifies a crucial factor of a more realistic experience for the future development of therapies using virtual reality. Furthermore, current graphical processing units’ unwritten law implies that a higher graphic fidelity correlates to lower frames per second, especially in standalone HMDs that lack high-end dedicated graphical processing power; a refresh rate measured constantly beyond 80 frames per second was therefore required by A.-M.G. as a means to ensure the mitigation of VRISE. With a significantly high cost for the resolution and embedding of additional environmental elements, this particular aspect led to a prolonged debate between the developers who were eager to harness more immersive elements. According to our safety-focused framework, the verdict was given by C.S. based on the motivation that “a high level of graphical fidelity immersion acquired at the price of refresh rate may not be suitable for self-exposure to VRET to preserve non-maleficence and mitigate adverse events”. Although no cybersickness mitigation method can entirely avoid this side effect of iVR exposure, as shown in Table 7, which results from neurologists’ literature reviews, A.-M.G. proposed a solution that led to a compromise consensus, resulting in the possibility of choosing „high” graphical quality that was found to be reasonable regarding consistency in frames per second. Therefore, the platform benefits from two Ginger iVR utilities freely available within the Unity Toolkit to ensure that user hardware is adaptable to cybersickness mitigation. First, the *DotEffect Prefab* tool establishes a common baseline (the frame of dots can be seen in the video abstract) in a virtual environment that will undoubtedly reduce the possible manifestations of simulation sickness. This concept allows the individual to focus on a stationary symbol in the simulated surroundings. Secondly, adding the *SingleNose* option perpetually uses an artificial nose in the user’s peripheral vision as a constant visual reminder anchor. As proven by the current literature, this implementation is likely to reduce the severity of cybersickness owing to a natural visual disturbance related to the positioning of one’s nose [100]. Concerning seizure mitigation, A.-M.G.’s extensive literature review has revealed a lack of evidence-based pharmacovigilance reports to bridge VR exposure to seizures; the SAFEvR ACT app is limited by the design of a red-derived chromatic theme.

### 4.2. Integrated Phobia Psychometric Questionnaires

This novel approach empowers users during their gamified therapeutic journey by providing an objective progress overview based on scoring using an auto-administered Visual Height Intolerance psychometric [101] questionnaire while also offering the possibility to report the emotional impact of exposure using the EmojiGrid embedding [102]. Furthermore, by allowing the possibility of reporting individual progress within a secure anonymized data-gathering frame, the application opens up the future perspectives of a continuous user feedback loop mechanism. If free access promotes general uptake, it could result in passively gathering significant amounts of statistically relevant homogenous variables within a unitary methodological approach. This perspective could overcome the small-effect size barrier of the current literature iVR reports by fast-tracking the development of evidence-based implementation guidelines.

Wireless, controller-free hand-tracking technology is a breakthrough, making it possible to submerge the patient into scenarios entirely while maintaining a high interactivity level.

Design studies on VRET systems under different conditions have focused on fear-based scenarios, design theory, and practical considerations [131]. For instance, Morton et al. introduced interoceptive and physical cues in the design process to immerse patients in the VRET system experience [132]. Additionally, conceptual frameworks such as those presented by Ulrich et al. address the much-needed systematic approach to research design critical for collecting homogenous data [133]. These frameworks ensure more than just the coherency and effectiveness of the systems developed by addressing the current methodological Wild West, as Birckhead refers to the subjective role of researchers pursuing the same outcomes in very different ways [134]. The heterogenicity of designs leads to inconsistent quantitative data gathering, making it impossible to achieve higher hierarchical evidence-based conclusions through systematic reviews and meta-analysis [135].

However, applying these frameworks to VRET systems faces many challenges because human psychology is subtle and requires targeted therapy. The environmental variables in VRET, such as exposure and exposure intensity and duration, must be tuned according to safety-designed therapy protocols and personal tolerance [136,137,138]. Presenting stimuli in a multimodal format in VRET also requires a delicate balance between sensory deprivation and overload. Simultaneously, optimal engagement and realism are needed for therapy to be effective. Technological advances have enabled a more sophisticated approach for designing VRET systems. Machine learning algorithms, for example, can adjust therapy settings in real time in response to physiological and behavioral feedback from the technology used to provide therapy. Moreover, biosensors can be combined with iVR systems to generate real-time data regarding a person’s physical responses, which are easily accessible for therapeutic monitoring and customization. The development of VRET follows a multidisciplinary approach. Understanding VRET requires knowledge of technology, design, neuroscience, psychology, and human behavior. Therefore, collaboration with psychologists is essential for overseeing VRET applications from the perspectives of ethics and therapy. In addition, extensive research is needed to understand the long-term impact of VRET applications on generating user behavior data.

Most research and population applications of VR-based interventions are limited by their impact over a short period following usage. If VRET is mainstream, therapeutic changes need to be understood. Shifting the design focus to ensure that VRET is user-friendly and safe allows user autonomy and control to be conducted ethically. These components include user-controlled feedback mechanisms of the system in which they can control an aspect of their exposure. Most importantly, the data generated from these system interactions should be used exclusively with the user in mind, as most VRET system patients are susceptible to exposure. Ultimately, with so many issues to consider for VRET to be mainstream and considering the lack of governmental policy support, we can only rely on the ability, experience, and inspiration in the mind of the designer, who may possess the finesse to combine all the components mentioned within a definitive, ready to be delivered, and free VRET product.

### 4.3. Smartphone-Delivered iVR Limitations That Motivated Cross-Platform HMD Availability

Although smartphones reduce the cost of iVR and simplify its design, making it viable for medical purposes, such as physical rehabilitation [139], the resulting iVR content may lack the immersive level needed to manage specialized phobias (Table 8).

Regarding specific phobia treatments, smartphone iVR limitations impact exposure therapy immersion and presence. Inferior graphical quality, higher latency, and a narrower field of view due to less powerful processors hinder realism [86]. Furthermore, limited user control and interactivity, and a less ergonomic design increase discomfort and reduce therapeutic efficacy [140]. These limitations affect the quality of interactions, are crucial for successful exposure therapy, and may not meet the depth required for specific phobias [141]. Although technological advancements promise to enhance smartphone iVR for treatment, more empirical research is needed to evaluate its effectiveness in the clinical setting. Reduced immersion and disrupted presence in smartphone iVR can affect psychological and physiological responses essential for effective exposure therapy outcomes.

### 4.4. Addressing Self-Discrepancy and Neuroticism Vulnerabilities in VRET

Although CBT and other forms of exposure are routinely used, there is a discrepancy between the expected extinction of exposure-induced symptoms of anxiety and the subsequent performance of exposure.

The efficacy of CBT VRET is often unpredictable owing to individual psychological constructs; self-discrepancy and neuroticism have a considerable impact on treatment success [142,143]. The integration of tailored psychotherapeutic approaches into the SAFEvR ACT framework was designed to improve the anxiety symptoms related to acrophobia and claustrophobia.

Anxiety and depression are empirically associated with self-discrepancy as a concept exploring the divergence between the existent self, the ideal self, and the so-called “ought self” [144]. A potent component is ideal self-discrepancy, which creates a markedly superior self-ideal to measure, increasing susceptibility and fear of perceived threats. It simultaneously increases neuroticism, which cascades the intensification of cognitive and affective variations, thus making exposure therapy less effective.

Moreover, self-discrepancy differs across cultures and has different actual, ideal, and ought configurations. More specifically, Asian populations are prone to vulnerabilities to developing anxiety amplification and not depression and, therefore, constitute a more vulnerable population when using the SAFEvR ACT app. We address this aspect by developing detailed oriental-derived theme levels to create transcultural acknowledgment and alleviate eventual anxiety. The possibility of instantly interrupting exposure, tutorials, and contextually triggered reflection psychotherapeutic voice interventions may also indirectly counter this issue.

This requires culturally sensitive tailoring of the exposure therapy. One of the values of safe ACT systems is that they offer a novel opportunity to use the diversity of virtual reality settings as part of therapy.

### 4.5. Rational Emotive Behavior Therapy Paradigm-Derived Contextually Triggered Audio Guidance

Audio-guidance prompts are based on the rapid identification and transformation of irrational beliefs to counter the presumed role of probable latency in promoting maladaptive emotional and physiological reactions during VRET. Thus, by countering vulnerable personality traits, rapid guidance may favor the user to avoid confrontation for vulnerable characteristics such as perfectionism, favoring incapacitating or guilt-determining interpretations of actions [145]. This perspective has already been proven to enhance mental health; the model is recommended to improve mental health within an athletic population, thus highlighting its versatility [146,147]. Chrysidis provided empirical evidence that this framework could help college football athletes develop intrinsic motivation and self-efficacy [148].

### 4.6. Personalised Challenges in Therapeutic VRET and the Role of Gamification

Interdisciplinary collaboration between technologists, clinicians, and neuroscientists is crucial for harnessing VRET’s potential. These collaborations yield comprehensive data relevant to patient experience and may generate guidelines for utilization as technology advances and are used in clinical neuroscience research.

The VRET system proposed by Oana Bălan et al. 2020 stands out for its unique features. It comprises nine interconnected modules, each offering patient profile setup, therapy scenes, audio-visual options, and physiological monitoring. What sets this system apart is the real-time biophysical signal viewing for therapists, enabling dynamic adjustment of the exposure scenario. The system also includes a reward system and a novel “emotion dimension”, which is set to be integrated into a machine learning-based emotion recognition exposure adaptation module [30,149].

Personalized objective inclusion involves protocols aimed at the patient’s specific therapeutic needs; such objectives should be formulated in a challenging yet achievable way to maximize the patient’s involvement with their subsequent feeling of accomplishment. Tailored tracking mechanisms seem necessary to reach these goals and offer real-time feedback on the process and the patient’s achievements. This should foster patient confidence by adhering to a small-step approach to ensure patient adaptability. In addition, sophisticated data analytics are crucial for detecting complex patterns embedded in data and helping to understand the meaning of these patterns. This approach may help identify the elements of virtual reality interactions that are more beneficial to patients, potentially assisting researchers in developing more focused VRET interventions. In summary, combining personalized goal setting and embedded tracking systems offers a more substantial benefit and value for therapeutic VR-based treatments. Specifically, this intervention model represents potential in gathering data to fine-tune the therapeutic approach, assuming that the patient is the central point based on motivation, structural cohesion, a flexible framework, and adaptability intent while utilizing VRET. Regardless, data-driven adjustment during application development, as proposed by Lai, could help maintain the efficacy of VRET over the treatment duration [150].

Gamification interventions for SAFEvR implementation have been developed to stimulate motivation, even in the most challenging scenarios. Novel aspects, such as determining voluntary exposure by confronting phobic stimuli, are integrated within behavioral conditional learning through the scoring system. This also allows users to benefit from the real-time tracking of maladaptive avoidance behaviors while building on progress through different utilizations.

Incorporating gamification promotes user engagement based on intrinsic motivation, ensuring that sufficient attention is paid to the therapeutic journey. When integrated with reward systems akin to video games, patients are prompted to remain dedicated to their therapy, which could facilitate better outcomes with the intervention. Our proof-of-concept of the SAFEvR protocol proves that the progression from traditional VRET to gamified VRET acts synergistically with the core psychotherapeutic foundations that make VRET effective when gaming elements align with exposure therapy’s fundamental goals and therapeutic ingredients.

The design of such systems must consider several aspects. For starters, the chosen VRET platform should be readily accessible to the cohort and relevant to current consumer hardware; according to Lindner, utilizing consumer hardware is readily available to patients and designed to improve their access and compliance [151,152]. The timeline of exposure therapy should be structured programmatically to maintain a graded confrontational experience that can be adjusted according to patient progress. The adaptation factor was congruent with inhibitory learning and extinction of the conditioned fear responses. In VR, a game can offer dynamic and personalized exposure experiences instead of a pre-designed static phobic trigger zone. The stimuli included in VRET must be provocative for the desired therapeutic response while engaging enough to incentivize repeat interactions. Adding virtual socialization to the equation could make the scenario cooperative or competitive. This could create a community and support not only for mental health and tech representatives but also among clinical patients. Moreover, the fidelity and specificity of gamified VRET environments and scenes should be congruent with patients’ actual fears. User-driven narratives might be used to achieve this, as they could incentivize participants to create stories around the exposure that could lead to enhanced outcomes. Adaptive algorithms can adjust exposure by considering patient performance, which could aid in ensuring a personalized result. Although the self-exposure VRET perspective would significantly unburden the current inability to perform psychological treatments, the presence of a psychotherapist to guide the paints cannot be replaced by artificial intelligence adaptative algorithms [153].

### 4.7. Limitations

The limitations of the current SAFEvR ACT study include its reliance on a narrow specialist user group of neuroscientists, mental health professionals, and tech developers for initial testing and the lack of need for extensive empirical validation. It also focuses solely on acrophobia and claustrophobia and depends on advanced iVR technology that may not be accessible to all users. Although deployment is done on a .app domain known for state-of-the-art security [154], there are still limitations, such as ongoing concerns about data security and ethical management.

To axiomatically follow the heuristic evaluation and maximize safety, the current stage of development allows only the self-experimentation of mental health professionals and tech developers. Although the current application version was received by the Research Ethics Committee for general population use, following the SAFEvR conceptual framework that focuses on safety since inception, we recommend that the application be utilized under mental health specialist supervision, especially for individuals with acrophobia.

### 4.8. Future Directions

Further collaborative partnerships are encouraged to improve the current stated limitations. The next step is IRB approval for the clinician-supervised pilot research study protocol before submission to the National Agency for Medicines and Medical Devices (ANMDM) of Romania approval regarding the SAFEvR ACT app for general utilization. Including psychotherapists within heuristic development represents a double-edged perspective if the same psychotherapists are future sub-investigators in future prevalidation assessments such as large-scale randomized control trials. Although unequivocally heuristic development specialists agreed that this possible technological bias risk probability is outweighed by the patient’s self-reported psychometric parameters within the iVR environment and collected automatically, there is a need for other specialists to be involved as future sub-investigators in future research that involves cohorts of patients.

Collaborative partnerships can help break through the current lack of momentum in developing specialist-shaped VRET applications and bring community-driven voluntary projects such as the MentalVerse.app closer to widespread use. National medical device authorization will follow IRB approval for clinician-supervised pilot studies. As a result of its adaptive nature, the platform has the potential to be further developed beyond acrophobia and claustrophobia, extended to procedural anxiety or relaxation, and besides phobias, through collaboration. Further development is needed for developing relaxation virtual environments and multilingual guided psychotherapy sessions to support conventional treatment of different mental health disorders, such as mild to moderate cases of depression, anxiety, and even eating disorders. Future works could benefit from the expected evolution of current machine learning algorithms to develop autonomous personalized treatment. Additionally, we envision future platforms that would receive the proper funding needed to create freely available iVR interventions to integrate biosensors, enabling real-time feedback to patients and their specialists in their homes as a means to a more efficient telemedicine perspective for mental health disorders. This would allow the VRET app to automatically prompt the patient towards more specific strategies during increasingly stressful scenarios concerning an individual reflection of anxiety perception as interpreted in real time by biosensors. Fine-tuning of such algorithms may facilitate effective desensitization based on their real-time physiological and behavioral responses. Still, although such platforms may offer the possibility to quantitatively gather large amounts of relevant data that may be helpful to progress in understanding and managing mental health disorders, the safety of the patient must stand as the cornerstone. Along with safety, privacy is a primary concern; therefore, data security is indispensable; research teams must continue developing protocols for securely handling data while addressing bias. Only when foolproof real-time data management is available, must participants be allowed to share their progression within the VRET app. Such endeavors necessitate a continuous iterative improvement-focused transdisciplinary dialogue involving patients as stakeholders [135].

## 5. Conclusions

Our transdisciplinary approach successfully delivered a functional application, transitioning from the proposed SAFEvR ACT framework to a proof-of-concept application freely available on the MentalVeRse.app. This demonstrates the potential of our framework as a viable blueprint for transdisciplinary synergy.

Developed following the SAFEvR ACT framework (Safe Adaptive-Gamified Free Exposure in Virtual Reality for Claustrophobia and Acrophobia Therapy), our research advances digital mental health by providing a free, scientifically grounded, and scalable VRET application for acrophobia and claustrophobia. The adaptive-gamified approach and the voluntary model used in this work highlight the potential for transdisciplinary collaboration in creating innovative and robust solutions to current digital mental health challenges.

The SAFEvR ACT MentalVerse.app heuristic development involved collaboration among bioethicists, neuroscientists, and tech developers. Our major finding is that gamification integration within VRET is a viable catalyst for blending transdisciplinary heuristic developments, bridging different specialist-derived perspectives towards new frontiers in treatment. This framework aims to enable effective self-exposure therapy by prioritizing safety as the core feature. If future research continues to develop free VRET self-exposure applications, the benefits could extend beyond alleviating the mental health burden of patients and practitioners.

With foolproof data collection security, this approach could passively gather significant quantitative data, which is essential for advancing our current limited VRET evidence base. Researchers are encouraged to pursue pathways for offering improved versions of such applications, starting from similar conceptual frameworks, to achieve widespread digital mental health adoption. We conclude with a call to action, inviting researchers to harness the transdisciplinary potential for critical novel approaches or fine-tune the current SAFEvR ACT application, freely downloadable at https://MentalVeRse.app.

## 6. Patents

The SAFEvR ACT App is available for modular collaborative development on the Association of Psychotherapy and Clinical Psychology websites: www.apipc.ro and https://MentalVeRse.app. The code will only be open source under reasonable request once participants’ account data-gathering protocols for security are completely externalized or deemed foolproof to eventual data breaches.

## Figures and Tables

**Table 1:** The heuristic approach to the VRET application development specialists’ team.

Role	Responsibilities
Two senior psychiatrists and bioethicists within The ”Socola” Institute of Psychiatry—the president and secretary of the Research Ethics Committee	Provide insights on ethical and safety issues, develop informed consent and emergency protocols, integrate access to professional help;
One psychiatrist, psychotherapist, and iVR researcher	Balances therapeutic efficacy with gamification and ensures engaging user experience (ThinkThank developer);
Four principal psychologists and psychotherapists at the Association of Integrative Psychotherapy and Clinical Psychology	Offer psychotherapeutic input for therapeutic desensitization and phobia exposure viability testing (ThinkThank developer);
One software developer and user interface designer	Enhances app usability and accessibility, ensures easy navigation for all users (ThinkThank developer);
One iVR Unity App Tech lead developer	Integrates framework suggestions into app updates and maintains technical soundness (ThinkThank developer);
One Neurologist, researcher	Adverse events’ mitigation, such as cybersickness/photosensitivity, aimed at minimizing iVR discomfort

**Table 2:** Thematic analysis of the SAFEvR protocol for developing a VRET application.

Principles: Broad Themes	Sub-Themes: Foundational Components	Iterative Improvements’ Specific Focus	Description of Iterative Improvements in the Manner of Implementation within the Application
**S**afety	Non-maleficence	Psychological safety	Ensure the app avoids physical and emotional harm and supports a sense of psychological safety, where users feel secure exploring and confronting their fears without judgment.
	Beneficence	Active benefit enhancement	Actively engage users in their therapeutic journey with personalized feedback loops highlighting progress, reinforcing achievements, and personalizing the journey.
	Confidentiality and privacy measures	Advanced data protection	Implement state-of-the-art encryption and anonymization techniques to ensure data protection beyond standard requirements. Offer users control over their data.
**A**daptive (gamification)	Personalization of treatment	Dynamic adjustment	Incorporate contextually triggered audio prompts to dynamically adjust real-time scenarios based on user responses, optimizing the therapeutic experience.
	Cultural sensitivity	Inclusive design	To make the app accessible and relevant worldwide, design it with a global perspective, including diverse cultural contexts, languages, and societal norms.
	User experience design	Immersive storytelling	Use narrative-driven scenarios, which increase engagement by placing the user in a story, making the therapeutic process more relatable and compelling.
	Usability	Intuitive interaction	Leverage advanced UX(User Experience)/UI (User Interface) principles to ensure that even users with minimal digital literacy can navigate the app effectively. Use natural gestures and voice commands for interaction.
**F**reely available and accessible **E**xposure	Evidence-based practice	Continuous learning system favoring iterative improvements	Integrate a system within the app that gathers feedback from aggregated, anonymized user data to improve therapeutic protocols and effectiveness over time.
	Autonomy in VRET apps	Empowered decision making	Give users more control over their therapy, including customizing the content, intensity, and duration of sessions.
	Distributive justice	Universal access	Ensure the app is free, optimized for low-bandwidth environments, and available on multiple platforms, including affordable iVR headsets such as Oculus Quest 2 HMD.

**Table 3:** State-of-the-art literature guidelines regarding VRET application development.

Safety—Ethical	Adaptive Gamification	Free (Accessible User Interface Design)	Psychotherapeutic Exposure
Informed consent as a treatment framework strategy [75]	Considering the impact of high-stress simulations on well-being [76]	Informed consent within the interface [77]	Personalization and adaptability [78,79]
Mitigating the risk of traumatizing or re-traumatizing participants [76]s	Personalization of the parameters of the therapy [80]	User autonomy [49,50,81]	Privacy compared to in vivo exposure Personalized plan specific for the client [79]
Moral and legal accountability [65,71] Data sovereignty [74]	Technological and ethical barriers [82]	Evidence-based design [78,79]	Psychotherapy Vr integration Autonomy Self-diagnosis Self-treatment Expectation bias [82]
User safety Professional oversight Accessibility [77,79,83,84]	Agency Trust Presence User-centered approach [85]	Transparency, usability, and trustworthiness through design [45,86]	iVR safe-check framework predictable pitfalls [87]
Privacy confidentiality [77,79,82]	VR-check framework also evaluates user motivation [87]	Intuitive application interfaces are usable without guidance [88]	Representation of the self in virtual environments [80]

**Table 4:** Aspects derived from ISO Ergonomics of human–system interaction, Part 820: Ergonomic guidance on interactions in immersive environments, including augmented reality and virtual reality [49].

Category	Consideration	Description
Interface design	Clarity and simplicity	Ensure the iVR VR interface is straightforward, with clear instructions and an intuitive layout.
	Accessibility	Incorporate features like adjustable text size, contrast settings, and audio cues to accommodate diverse user needs.
User interaction	Responsive controls	Controls should be responsive and easy to manage, especially under stress.
	Feedback systems	Implement immediate feedback through visual, auditory, or haptic cues to guide and reassure users.
Customization	Adaptive difficulty	Adjust the intensity of exposure based on real-time feedback and physiological indicators to keep therapy within comfortable limits.
	Personalization	Allow users to personalize settings like environmental details and types of stimuli.
Therapeutic alignment	Progress tracking	Features to monitor user progress over sessions should be integrated, helping to adjust therapy as needed.
	Evidence-based design	The therapy scenarios should be designed based on clinical research to ensure effectiveness.
User education	Guidance and support	Provide in-app educational resources about the contextual therapy process and suggest anxiety management tips.
	Real-time assistance	Offer voice prompts or real-time human voice audio support that is accessible during therapy sessions to aid users contextually.
User satisfaction	Engagement features	Integrate elements like gamification and rewards to motivate and engage users.
	Community integration	Enable features that connect users with peers to foster support networks and reduce feelings of isolation.

**Table 5:** Safe—Ethical implementations.

Ethical Principle	Description
Informed consent	Before VRET, participants must be fully informed about the therapy’s nature, risks, benefits, and rights within the virtual environment [90]
Confidentiality and privacy	Patient data and interactions within the virtual environment must be secure and protected to maintain trust and confidentiality [82].
Beneficence and Non-maleficence	Practitioners must ensure iVR interventions benefit patients and minimize harm, including monitoring for adverse effects such as VR-induced symptoms and effects (VRISE) [91,92,93,94].
Autonomy and respect	Participants should have the autonomy to make informed choices about their treatment, including options for customization and control [34,95].
Ethical review	To address potential ethical concerns, a thorough ethical review of VRET protocols and interventions should be conducted [82].
Cultural sensitivity	Virtual environments and exposure scenarios should be adapted to be culturally sensitive and respectful [96].
Continuous monitoring and feedback	It is crucial to monitor participant’s progress regularly, collect feedback, and adapt protocols based on responses [97,98,99]
Cybersickness mitigation	DotEffect Prefab and Singlenose from GingerVr integration [100]
Data security	User data and interactions within the iVR environment must be secured [97]
Visual height intolerance questionnaire (quantitative data)	Integration within the application—possibility to measure progress [101]
Emojigrid (qualitative data)	Adding the possibility of reporting the affective impact of exposure [102]
Weightless, Marconi Union	This soundtrack proved to lower anxiety by 65%, comparable to therapeutic massage—therefore, a licensed version was introduced as an option to anchor the user within a therapeutic environment [103,104,105].

**Table 6:** **A**daptive gamification serving as a synergic integrator for the SAFEvR ACT application.

Aspect	Implementation Detail	Safety	Adaptive Gamification	Accessibility/ Usability	Exposure Psychotherapy Integration
**Scoring System**	The scoring system manages multiple games, allowing users to select levels from a menu. Users activate games by interacting with a green terminal. A red terminal is available to stop the game and save scores. The system uses abstract *GameBase* class methods for flexibility.	Ethical compliance and risk minimization are ensured by providing clear instructions and safety exits in the iVR environment. Users can stop the game anytime for safety, with non-saved scores as a precaution against distress.	Scores adapt based on user actions and performance, ensuring personalized challenges. Unique gems and “*look down”* areas adjust based on user progression, balancing engagement with therapeutic exposure levels.	Menu and terminal interfaces are designed for straightforward navigation, enhancing user comfort and interaction with the game mechanics. Force-stop options ensure user control over the experience, improving usability.	Games integrate exposure therapy techniques, like “*LookDownGam*e” for acrophobia and “*RoomShrinkingGame”* for claustrophobia, providing controlled exposure in a gamified context. Tutorials were added for user guidance and therapeutic efficacy. The user behavior triggers rational emotive behavioral therapy-derived narrative audio prompts
**Gem** **Collecting Game**	Spawns blue gems guiding users towards an objective, with unique gems offering higher points based on color intensity. The game encourages exploration and interaction within the iVR environment.	Safety mechanisms ensure the exploration does not induce excessive discomfort or risk, with clear pathways and escape options.	The game dynamically introduces unique gems based on user progress, adapting the challenge to maintain engagement and therapeutic goals.	Accessible design features ensure that users with varying levels of iVR familiarity can engage with the game, and tutorials are provided for guidance.	Synergistically facilitates therapeutic exposure by motivating distractibility while immersing in the exploration through interaction with the environment within controlled parameters.
**Look Down Game**	Utilizes signs to demarcate "look down" areas, rewarding or deducting points based on the user’s gaze direction. It employs ray casts to detect gaze direction, optimizing it for when users look down.	It incorporates feedback mechanisms to mitigate adverse events, such as point deduction for avoiding therapeutic views and ensuring safety while promoting exposure therapy principles.	The challenge is adapted based on the user’s ability to confront fear-inducing views, offering points as immediate, adaptive feedback for therapeutic actions.	It is designed to be intuitive, with signs indicating interactive areas and the consequences of actions, enhancing the user’s ability to navigate and understand game mechanics.	It directly integrates exposure therapy by rewarding users for confronting fear-inducing stimuli and making real-time adjustments to ensure a therapeutic level of challenge.
**Room Shrinking Game**	Targets claustrophobia by simulating enclosing walls. Points are awarded as walls close in, with a pause option for user control. Incorporates a “*NoWallPeeking”* script to prevent cheating.	Ethical considerations include a pause feature for user comfort and control and safety scripts like “*NoWallPeeking”* to ensure users remain within therapeutic boundaries.	The game’s pace and wall movement adapt to user performance and comfort level, offering a personalized therapeutic challenge.	The game’s design prioritizes user comfort with clear instructions and the ability to pause, enhancing the overall usability of the exposure therapy tool.	They are designed explicitly for claustrophobia therapy, with controlled exposure to narrowing spaces and mechanisms for user control and comfort during sessions.

**Table 7:** Literature overview of pharmacological and non-pharmacological management of cybersickness.

VRISE	Non-Pharmacological Interventions	Pharmacological Interventions	Practical Considerations
Cybersickness/VIMS Symptoms: Headache Nausea Dizziness	Gradually acclimatize individuals to iVR environments, utilize anti-motion sickness techniques, and implement short, repeated iVR exposure sessions [116].	Employ antihistamines such as dimenhydrinate, anti-nausea medications like ondansetron, and anticholinergics including scopolamine [117,118].	Monitor and adjust the duration and intensity of iVR exposure to enhance comfort and reduce symptoms [119].
Temporary disorientation and nausea are specific to anxiety disorders	Apply cognitive–behavioral strategies and anxiety management techniques and establish personalized iVR exposure limits [120]	Use benzodiazepines such as lorazepam for acute anxiety management and SSRIs for long-term treatment of anxiety disorders [121,122]	Conduct careful patient selection for iVR therapy, taking preexisting anxiety disorders into account.
Exacerbation of vestibular symptoms: vertigo, imbalance	Monitor and adjust the intensity of iVR rehabilitation exercises combined with traditional vestibular rehabilitation therapy [123].	Administer betahistine for vertigo management and vestibular suppressants like meclizine for acute exacerbations [124]	Closely monitor symptoms, adjusting or discontinuing iVR as necessary [125].
Visual disturbances and sense of unreality	Ensure proper iVR headset adjustment, limit session duration, and gradually expose users to iVR settings. Visual rest and exercises may also be beneficial [126].	Manage underlying anxiety or phobias with SSRIs or SNRIs; no specific pharmacological treatment for visual disturbances [127]	Adjust iVR technology settings and session timing for individual comfort and safety.
Increased physiological responses: heart rate, sweating	Incorporate relaxation techniques before and after iVR sessions and monitor physiological responses to adjust therapy intensity [128].	Use beta-blockers like propranolol for managing physiological symptoms during high-anxiety situations [129].	Be aware of distress signs, and incorporate breaks and relaxation for well-being [130].

**Table 8:** Overview of smartphone iVR limitations regarding VRET [83,86,140].

Category	Smartphone iVR Limitations	Impact on Exposure Therapy
Immersion and presence	Lower visual and audio fidelity.	Reduced immersion affects psychological and physiological responses, which are crucial for the efficacy of exposure therapy.
Technological limitations	Inferior graphical quality due to less powerful processors Higher latency issues The narrower field of view.	Limits realism needed for effective therapy Disrupts sense of presence and can induce motion sickness Reduces peripheral vision, decreasing environmental realism.
User control and interactivity	Limited tracking precision Less sophisticated user interfaces.	It affects the quality of interactions within the virtual environment, crucial for controlled exposure. It also restricts the ability to tailor and adjust environments dynamically.
Safety and comfort	Generally less ergonomic Higher risk of simulator sickness.	Decreases comfort during extended sessions, which are common in therapy Increased discomfort and disorientation could exacerbate phobia symptoms.
Clinical efficacy and application	A sufficiently immersive experience for deep therapeutic interventions may be needed, but more work is required to adapt it for controlled clinical use.	May not meet the depth of therapy required for severe phobias Therapists need more flexibility to fine-tune environments to therapy goals.
Future directions	Potential improvements with advances in technology and content creation There is a need for more empirical research comparing outcomes.	Technological advancements could enhance the viability of smartphone iVR for exposure therapy Further studies are necessary to understand the effectiveness in clinical settings fully.

## Data Availability

The SAFEvR ACT application is freely available to download at https://MentalVerse.app, while the code will only be available by request from the corresponding author until fool-proof user gathered data security can be guaranteed.

## Supplementary Materials

The following supporting information can be downloaded at https://www.mdpi.com/article/10.3390/brainsci14070651/s1, Table S1: User accessibility interface implementations; Table S2: Psychotherapist voice guidance prompts triggered by the user’s contextual behavior. Video S1: SAFEvR ACT MentalVerse application.
