# Supplementary material for: SAFEvR MentalVeRse.app: Development of a Free Immersive Virtual Reality Exposure Therapy for Acrophobia and Claustrophobia
Source: Brain Sci. 2024 Jun 27;14(7):651. doi: 10.3390/brainsci14070651 (PMC11274658; doi:10.3390/brainsci14070651)
Supplement: Supplementary file 1 [file brainsci-14-00651-s001.zip › Table S1. User accessibility interface implementations and Table S2..pdf]

Table S1. User accessibility interface implementations

Section	Feature Description	Implementation Details
Settings Menu	Manages user preferences.	- Saves and loads user settings using serialization. Stored settings persist across scenes via the *MainManager*.
In-Game Menu	Accessible menu in the training environment.	- Appears when the user rotates their left hand to a specific angle, enabling easy repositioning and visibility adjustments. Includes exit and save buttons.
Locomotion System	Facilitates movement within the virtual environment.	- Incorporates *XR* (Extended Reality) *Toolkit* components for various locomotion methods: Teleportation Provider, Continuous Turn Provider (right hand), and Continuous Move Provider (left hand).<br>- Teleportation Controller script handles the joystick input for teleportation.
Climbing System	Allows climbing as an alternative traversal method.	- Managed by the *ClimbingProvider* class derived from *LocomotionProvider*, checking user eligibility for climbing. GravityToggler class manages gravity effects during climbing. Tested using an actual iVR headset for performance validation, optimized for Oculus Quest 2.
Fall Prevention	Mechanism to maintain the realism of falling consequences.	- FallingDetection class detects falls and manages user recovery without using invisible walls to maintain immersion and prevent navigation issues.
Scoring System	Optional system rewarding navigation and facing fears.	- Managed by the *ScoringSystem*, which tracks scores across different games and levels. *GemCollectingGame* and *LookDownGame* add interactive elements and challenges and are integrated using the *GameBase* abstract class.
Safe Zone Button	Quick access button for exiting overwhelming fear-inducing scenarios.	- The repurposed menu button on the player's left-hand acts as a shortcut to teleport back to the main menu to exit from uncomfortable situations immediately.
Scenes	Diverse environments with different challenges.	- Environments such as *Canyon*, *CatRescue*, *ChineseHouses*, *RopeBridge*, and ShrinkingRoom, each with specific elements to challenge and engage the player in various phobia-related scenarios.
Localization	Supports multiple languages.	- Utilizes *Unity*'s localization system with *StringTables* for different locales, ensuring all UI elements adjust to the selected language. Questionnaires require separate JSON (JavaScript Object Notation) files for different languages.

Table S2. Psychotherapist voice guidance prompts triggered by user's contextual behavior

Category	Audio guidance (both male and female voice, English and Romanian)	Triggers
Enhancing engagement and skill acquisition	"*Remember, you're in a safe space. Let's take a slow, deep breath together. Inhale slowly through your nose, then exhale through your mouth. Feel your feet grounded in this virtual world.*"	When the user accesses the SAFE button.
Providing real-time support	"*If you're feeling anxious, it's okay. Let's pause for a moment. Focus on identifying five things you can see, four things you can touch, three things you can hear, two things you can smell, and one thing you can taste within virtual space.* "	Recognize distress and promote attention shift.
Tailoring therapy to individual needs	"*You're doing great navigating through this scenario. If this feels overwhelming, we can adjust the intensity at any time. Just let me know how you're feeling, and we'll proceed at a pace that's comfortable for you.*"	Fast progress – when achieving more than 80% of the possible maximum score
Facilitating self-efficacy and autonomy	"*You've encountered a challenging situation. Let's use the strategies we've practiced. Start by reminding yourself that you have the skills to navigate this. What strategy would you recommend to someone you love to use in this situation? (three seconds pause) – now try to apply that strategy yourself.*"	Repeating the same level without progressing
Multilingual informed consent	This includes an introduction, the purpose of the SAFEvR ACT, potential benefits and risks to consider, a feedback option, data anonymity and security, and a final statement.	Before accessing the application, written and audio formats for accesibility.
